# Superior vena cava syndrome revealing a Behçet’s disease

**DOI:** 10.1186/s12959-015-0039-z

**Published:** 2015-01-31

**Authors:** Simon Antoine Sarr, Pape Diadie Fall, Mouhamadou Chérif Mboup, Khadidiatou Dia, Malick Bodian, Modou Jobe

**Affiliations:** Service de cardiologie, CHU Aristide Le Dantec, Dakar, Sénégal; Service de cardiologie, Hôpital Principal de Dakar, Dakar, Sénégal

**Keywords:** Superior vena cava syndrome, Thrombosis, Behçet, Dakar

## Abstract

**Introduction:**

Behçet’s disease (BD) is a rare vasculitis in sub-Saharan Africa. Vascular thrombosis, especially venous, is common in this condition and also constitutes a basic diagnostic criterion. Its affection of the superior vena cava is rather rare with only a few cases described in the literature.

**Case report:**

A 42-year-old male patient was seen at consultation presenting with a pulsatile, warm and slightly painful right latero-cervical swelling extending to the supraclavicular fossa with the presence of collateral venous circulation for three weeks prior to presentation associated with a mild headache. There were oral and genital ulcerations and erythematous skin lesions associated with a history of inflammatory recurrent arthralgia. Chest computed tomo-angiography showed cruoric internal jugular vein thrombosis extending to the superior vena cava with significant venous collateral circulation. The patient was treated with prednisolone (1 mg/kg/day) and colchicine (2 mg/day), as well as anticoagulation with heparin and vitamin K antagonist (Acenocoumarol) with regular INR monitoring. Clinical evolution was favorable during hospitalization, with residual discrete right supraclavicular swelling. There was no bleeding associated with anticoagulants use.

**Conclusion:**

The case stresses the importance of maintaining a high degree of suspicion for Behçet’s disease in all cases of venous thrombosis.

## Introduction

Behçet’s disease (BD) is a multisystemic inflammatory disease characterized by recurrent oral ulcers, genital ulcers, and uveitis. It is a rare vasculitis in sub-Saharan Africa and is more common in the Mediterranean region. Vascular thrombosis, especially venous, is common in this condition and also constitutes a basic diagnostic criterion [1]. Its affection of the superior vena cava is rather rare with only a few cases described in the literature [2,3]. We present the case of a patient with Behçet’s disease which was discovered during a superior vena cava syndrome.

## Case report

A 42-year-old male patient was seen at consultation with pulsatile, lateral neck pain for three weeks prior to presentation associated with a mild headache. This pain, described as heaviness, was sometimes located in the mid-thoracic region, exacerbated by a slight dry cough and associated with a low-grade fever and sometimes with an exertional dyspnea. There was no associated hemoptysis, vomiting, chills, sweating or dizziness. His past medical history was unremarkable apart from recurrence of oral and genital ulcers as well as inflammatory arthralgia.

**Figure 1:**
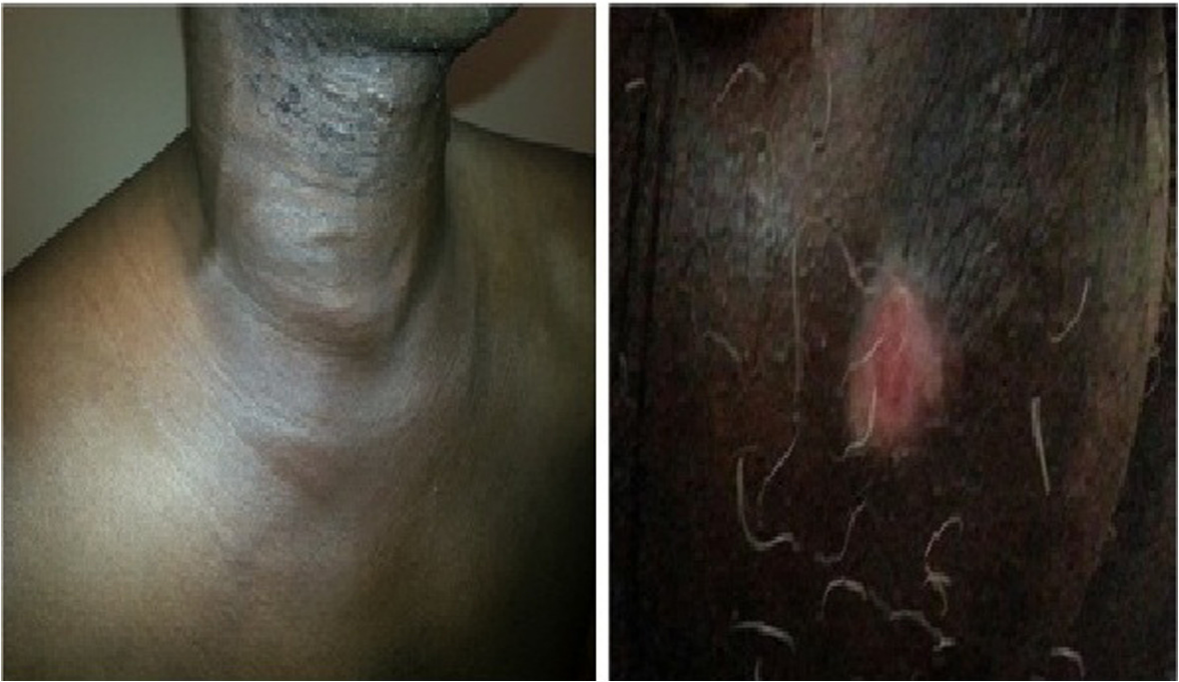
**Edema and significant collateral venous circulation (left), scrotal ulcer in recovery phase (right).**

On admission, clinical examination revealed a hyperthermia at 38.3°C but was hemodynamically stable. There was a warm, slightly painful, right latero-cervical swelling extending to the supraclavicular fossa. There was evident collateral venous circulation (Figure 1). The lung fields were clear, and there were no palpable lymph nodes and no signs of phlebitis. Further examination revealed healing scrotal ulcers and oral ulcerations as well as erythematous skin lesions.

Ophthalmologic examination was unremarkable. Laboratory tests showed a microcytic anemia with a hemoglobin concentration of 9.6 g/dl, with a normal white blood cell count of 9,100/mm^3^. Admission electrocardiogram noted a regular sinus tachycardia. Doppler transthoracic echocardiography noted a slight circumferential pericardial effusion with no sign of pulmonary hypertension or intracavitary thrombus.

**Figure 2:**
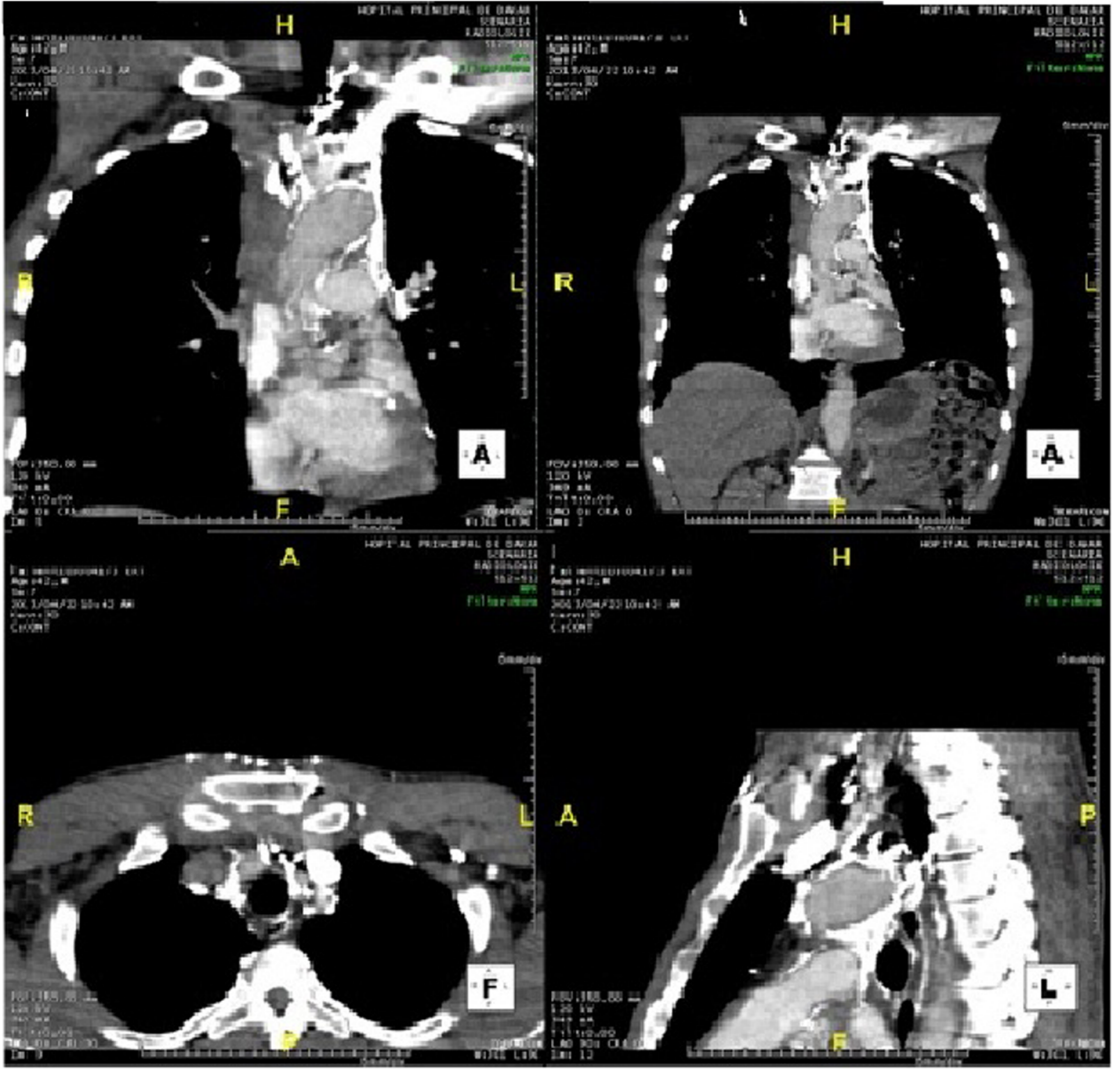
**Thoracic CT angiography showing an internal jugular and superior vena thrombosis.**

Chest computed tomo-angiography showed cruoric internal jugular vein thrombosis extending to the superior vena cava with significant venous collateral circulation. There was no hilar or mediastinal lymphadenopathy, neither was there any lung parenchymal abnormality (Figure 2). The abdominopelvic ultrasound was normal.

The patient was treated with prednisolone (1 mg/kg/day) and colchicine (2 mg per day), as well as anticoagulation with heparin and a vitamin K antagonist (Acenocoumarol), with regular INR monitoring. Clinical evolution was favorable during hospitalization, with residual discrete right supraclavicular swelling. There was no accident associated with anticoagulants.

## Discussion

BD is a systemic, inflammatory, immune dysfunction, which is chronic and relapsing. It is characterized clinically by recurrent oral and genital ulcers, suggestive skin lesions (pseudo-folliculitis, reaction at injection sites etc.), arthritis, uveitis and retinal vasculitis, central neurological impairment, venous thrombosis and intestinal lesions. There is no pathognomonic feature of BD and the diagnosis is based on clinical criteria such as those proposed by the International Study Group for Behçet’s Disease, and more recently, the International Team for the Revision of the International Criteria for Behçet’s Disease [4,5]. The diagnosis in our patient was clinical, and was based on recurrent genital and oral ulcerations and the presence erythematous skin lesions using the criteria drawn up by the International Study Group for Behçet’s Disease.

The disease is more prevalent in the Mediterranean region, the Middle East and in Japan [6]. It is rare in sub-Saharan Africa with no data available on its actual prevalence [7]. However, reports from the continent suggest that lack of awareness of the disease suggest that it is possibly under-diagnosed [8].

The vascular manifestations are seen in 10-40% of cases of BD [4] and it is predominantly venous which represents 80 to 90% of cases [9]. Vascular thrombosis are common found in 19.7% in the Spanish registry according to Rodriguez- Carballeira et al. who also found that men had higher prevalence of ocular involvement and venous thrombosis (52.5% vs. 39.2%, p = 0.004 and 26.3% vs. 9.6%, p < 0.001, respectively) [10]. Ideguchi also found a male predominance of vascular manifestations in BD (62%), with venous more than arterial (81% vs 31%) [11].

The common anatomical substratum of all these affections is vasculitis predominantly venous. The pathophysiology of BD remains largely obscure. It is well established that it involves infectious factors and abnormal immune reaction, both innate and adaptive raising questions about the nature of the BD: auto-inflammatory or autoimmune or rather at the crossroads of the two [9].

Their thoracic vascular location is well known. Thrombosis of the superior vena cava is possible in Behçet’s disease and represents 2.5% of cases [6]. However, it is rarely the presenting feature [12].

Superior vena cava thrombosis may be primary or secondary to an extension of axillary or subclavian vein thrombosis. It usually occurs a few years after the mucocutaneous signs of the disease. Rarely, it is the inaugural manifestation preceding the other events. Clinically, it may be latent, well tolerated, with a silent evolution, or it may have a rather aggressive manifestation characterized by chest pain, headache, bilateral papilledema and fever. It is possible to have, associated with the clinical features serous effusion as is the case in our patient with a pericardial effusion. Its very nature is sometimes chylous.

Patients with Behçet’s disease respond well to standard steroid and colchicine treatment [13] and there was a good evolution with our patient. Anticoagulation was given to treat vascular thrombosis.

Imaging plays a fundamental role in the diagnosis. This includes chest radiograph but especially computed tomography and magnetic resonance imaging [6].

## Conclusion

Although infrequent in our regions, Behçet’s disease should be included in the search for etiological venous thrombosis. Moreover, the orientating signs are quite specific guidance.

## Consent

Written informed consent was obtained from the patient for publication of this Case report and any accompanying images. A copy of the written consent is available for review by the Editor-in-Chief of this journal.
